# Muscle blood flow is independent of conduit artery diameter following prior vasodilation in males

**DOI:** 10.14814/phy2.14698

**Published:** 2021-01-11

**Authors:** Timothy R. Rotarius, Jakob D. Lauver, John R. Thistlethwaite, Barry W. Scheuermann

**Affiliations:** ^1^ Department of Exercise Science and Athletic Training Adrian College Adrian MI USA; ^2^ Department of Kinesiology Coastal Carolina University Conway SC USA; ^3^ Department of Health and Sport Studies Wittenberg University Springfield OH USA; ^4^ School of Exercise and Rehabilitation Sciences University of Toledo Toledo OH USA

**Keywords:** exercise hyperemia, muscle pump, vascular tone, vasodilation

## Abstract

At the onset of exercise in humans, muscle blood flow (MBF) increases to a new steady‐state that closely matches the metabolic demand of exercise. This increase has been attributed to “contraction‐induced vasodilation,” comprised of the skeletal muscle pump and rapid vasodilatory mechanisms. While most research in this area has focused on forearm blood flow (FBF) and vascular conductance, it is possible that separating FBF into diameter and blood velocity can provide more useful information on MBF regulation downstream of the conduit artery. Therefore, we attempted to dissociate the matching of oxygen delivery and oxygen demand by administering glyceryl tri‐nitrate (GTN) prior to handgrip exercise. Eight healthy males (29 ± 9 years) performed two trials consisting of two bouts of rhythmic handgrip exercise (30 contractions·min^−1^ at 5% of maximum) for 6 min, one for each control and GTN (0.4 mg sublingual) condition. Administration of GTN resulted in a 12% increase in resting brachial artery diameter that persisted throughout the duration of exercise (CON: 0.50 ± 0.01 cm; GTN: 0.56 ± 0.01 cm, *p* < 0.05). Resting FBF was greater following GTN administration compared to control (*p* < 0.05); however, differences in FBF disappeared following the onset of muscle contractions. Our results indicate that the matching of FBF to oxygen demand during exercise is not affected by prior vasodilation, so that any over‐perfusion is corrected at the onset of exercise. Additionally, our findings provide further evidence that the regulation of vascular tone within the microvasculature is independent of the conduit artery diameter.

## Graphical abstract

From an elevated resting blood flow (GTN administration), the addition of exercise does not further increase muscle blood flow during rhythmic, handgrip exercise. However, there are significant differences in retrograde blood velocity between GTN and CON. This suggests that downstream resistance in the microvasculature is increased despite a significantly greater blood flow upstream (brachial artery).

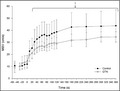

**What is the central question of this study?**

Control of muscle blood flow is a complex system that may differ between the macro‐ and microvasculatures. The role of the conduit artery as opposed to downstream vasculature in determining muscle blood flow has been scarcely explored.

**What are the main finding and its importance?**

Glyceryl tri‐nitrate (GTN) administration provokes an increase in FBF at rest, but not during light handgrip exercise compared to control. Additionally, the change in FBF during muscle contractions is lower with GTN administration, yet the absolute blood flow is unchanged between conditions. The latter suggests control of blood flow in the microvasculature is independent of vascular tone within the conduit artery.

## 1 INTRODUCTION

Vascular tone reflects the balance between centrally mediated sympathetic vasoconstriction and the release of humoral vasodilators in the periphery (Buckwalter & Clifford, 2001). At the onset of exercise, muscle blood flow (MBF) increases toward a new steady‐state resulting in a close coupling between oxygen delivery and the metabolic demand of the active muscle (Andersen & Saltin, 1985; Clifford, 2007; Mortensen & Saltin, 2014; Rosenmeier et al., 2004). The tight coupling of oxygen delivery to the demand for oxygen is very important under many conditions, as any mismatch between blood flow and metabolic demand is often observed in diseased states, including type 2 diabetes mellitus (Laakso et al., 1992). Additionally, early fatigue may occur during physical activities ranging from activities of daily living to high‐intensity sport performances should muscle blood flow be insufficient to meet the demands of the activity.

Recently, (Shepherd et al., 2016) concluded that muscle blood flow and oxygen delivery could be uncoupled from metabolic demand during forearm exercise by a continuous infusion of adenosine triphosphate (ATP), a potent vasodilator acting on the purinergic P_2γ_ receptors on endothelial cells and vascular smooth muscle (Kim et al., 2015; MacDonald et al., 1998). The infusion of ATP resulted in a significant increase in forearm MBF under resting conditions that was further increased with the addition of moderate‐intensity handgrip exercise (Shepherd et al., 2016). Since the increase in forearm MBF during exercise was considerably higher following ATP infusion compared to control conditions, these investigators argued that this was evidence of an uncoupling (i.e. an over‐perfusion) of blood flow and oxygen delivery relative to the metabolic demands of the exercising muscle (Shepherd et al., 2016). Although the magnitude of the forearm blood flow response to handgrip exercise was similar between ATP infusion and control conditions, the increase in forearm blood flow with ATP infusion from an already elevated blood flow indicated that there was an additional vasodilatory response with exercise that was not evident under control conditions. Our current understanding would argue that if O_2_ delivery were the fundamental determinant for blood flow during exercise, then enhancing blood flow prior to exercise would attenuate one or more of the redundant mechanisms for eliciting vasodilation in the active muscle. For Shepherd and associates (Shepherd et al., 2016), the specific mechanism underlying the increase in forearm blood flow with exercise following ATP infusion was not determined, but it was suggested that vasoactive substances such as nitric oxide (NO), prostaglandins, and/or endogenous ATP may have been released during exercise causing further vasodilation or the mechanical effects of muscle contraction may have contributed to the higher blood flow (Shepherd et al., 2016). Regardless of the mechanism, the apparent uncoupling of muscle blood flow from the metabolic demand of the active skeletal muscle challenges our current understanding of the regulation of muscle blood flow and warrants further investigation.

Recently, (Ranadive et al., 2019) investigated the effects of local vasodilator infusion and the superimposition of exercise on the muscle blood flow response. By infusing both adenosine and sodium nitroprusside, a NO donor, the authors were able to elevate the baseline MBF response. In addition, the superimposition of exercise on vasodilator infusion, and thereby an elevated baseline blood flow, resulted in no further increases in the hyperemic response (Ranadive et al., 2019). These authors concluded that other vasodilatory substances involved in the regulation of vascular tone contributed to the lack of further hyperemic or vasodilatory responses (Ranadive et al., 2019). However, it should be noted that this study utilized local infusion of vasodilators, which affected the local vasculature, whereas measurements of brachial arterial diameter and blood velocity may not experience the same effect to the vasodilatory drug. Thus, the administration of a systemic vasodilator like glyceryl tri‐nitrate (GTN) may provide further insight into the complex regulation of systemic vascular control.

Several investigators have argued that the skeletal muscle pump contributes to the increase in MBF at the onset of exercise as a result of rhythmical muscle contractions creating a lower pressure in the venous circulation during relaxation thereby increasing the pressure gradient across the muscle vasculature (Hamann et al., 2003; Remensnyder et al., 1962; Rosenmeier et al., 2004). While the muscle pump has been considered by many to be an important determinant of MBF at the onset of exercise, the findings of several studies performed using animal models and humans has led many investigators to question the significance of the muscle in the regulation of MBF (for a review see Joyner & Casey, 2015). Additionally, contraction intensity (% MVC) appears to play a role in the effectiveness of the muscle pump. For example, (Hamann et al., 2003) hypothesized that an infusion of adenosine to induce vasodilation in the muscle vasculature prior to the onset of exercise would lead to an increase in MBF due to improved actions of the muscle pump. Similar to the findings of Shepherd and colleagues (Shepherd et al., 2016), MBF was elevated at rest following the infusion of adenosine; however, in contrast, Hamann and colleagues (Hamann et al., 2003) observed a significant decrease in MBF at the onset of exercise leading these investigators to conclude that any increase in the pressure gradient across the muscle vasculature as a result of the adenosine‐induced vasodilation did not enhance the effects of the muscle pump. In contrast, Lutjemeier and colleagues (Lutjemeier et al., 2005) found that MBF was enhanced by the muscle pump following light intensity (4–5% MVC), while work rates greater than 5% MVC, up to 75% peak work rate showed no systematic enhancement of blood flow. Interestingly, (Hamann et al., 2003) showed that MBF was still elevated during exercise following the infusion of adenosine compared to control conditions, indicating that there may have been an uncoupling of MBF and the metabolic demands of the exercising muscle (Hamann et al., 2003). Thus, it appears as though the infusion of a vasoactive substance that results in appreciable vasodilation prior to the onset exercise may lead to a mismatch (i.e. over‐perfusion) of the exercising muscle during exercise. Nakamura and associates (Nakamura et al., 1997) have previously shown that the vasodilation of large conduit arteries may play a role in the adjustments of blood flow to downstream peripheral tissue. Thus, it is possible that change in conduit artery diameter reflect, in some portion, changes in downstream vascular endothelial function, and should be considered when measuring muscle blood flow during exercise. However, the extent that an increase in conduit artery diameter contributes to the mismatch between MBF and metabolic demands under conditions of prior vasodilation has not been established, but is critical to our understanding of the regulation of MBF during exercise.

Thus, the purpose of this study was to measure changes in brachial artery diameter and MBF at rest and during handgrip exercise during control compared to prior vasodilation using glyceryl tri‐nitrate administration. GTN, a known potent vasodilator, donates NO when metabolized by aldehyde dehydrogenase II which then acts directly on the vascular smooth muscle causing relaxation and vasodilation. We hypothesized that MBF would be higher at rest following the administration of GTN due to an increase in brachial arterial diameter and that the addition of handgrip exercise would lead to further vasodilation and increase in blood flow, similar to the results of Shepherd et al. (2016). These findings would be consistent with the uncoupling of blood flow and oxygen delivery from the metabolic requirements adding support to the recent findings demonstrating a dissociation between MBF and the metabolic demand of the exercising muscle.

## 2 METHODS

### 2.1 Ethical approval

Each subject was informed of all testing procedures, as well as the risks and discomforts associated with participating in the study. Subjects were given an opportunity to ask questions prior to providing written informed consent. This study complied with the standards set by the Declaration of Helsinki and was approved by the Human Research Protection Program and Institutional Review Boards at the University of Toledo.

### 2.2 Subjects

Nine healthy individuals (age range 18–40 years) volunteered to participate in this study. Subjects were excluded from this study if they had any known cardiovascular or metabolic diseases, or if they were currently prescribed sildenafil citrate, tadalafil, or other medications that lower blood pressure. Any individuals that experienced frequent migraines (1–2 times month) were also excluded from participation in the study. Subjects were considered recreationally active based on responses to a physical activity questionnaire. Subjects were instructed to refrain from any moderate to vigorous physical activity, while enrolled in the study and to avoid consuming caffeinated beverages on any of the study days.

### 2.3 Preliminary session

Each subject participated in a familiarization‐preliminary data collection session that included anthropometric measurements, maximal handgrip strength, and screening for clearly identifiable borders of the brachial artery wall. Subjects were excluded from the study if an image of clearly identifiable borders could not be obtained at rest or maintained during exercise.

Subjects were asked to perform three maximal voluntary contractions (MVC) of the forearm muscles to determine the weight to be used during the dynamic handgrip exercise. Subjects gripped a standard handgrip dynamometer (TKK5401 Grip‐D, Takei Scientific Instruments Co.) and maximally contracted for 5 s, after which the value on the digital display was recorded. Subjects were given 2 min of rest between each MVC and the average of the three trials was calculated as each subject's MVC.

Subjects were asked to lay down in the supine position with their dominant arm outstretched to approximately a 90° angle for brachial artery screening. Subjects that had identifiable brachial artery walls underwent a preliminary GTN administration procedure. Since GTN has a relatively short half‐life (Chen & Stamler, 2006; Corretti et al., 1995), each subject was administered GTN and brachial artery diameter was measured continuously until the increase in brachial artery diameter reached a new steady‐state. Brachial artery diameter and the time‐to‐peak vasodilation were recorded and used to determine the timing of measurements during the subsequent tests involving GTN administration.

### 2.4 Experimental protocol

Following the familiarization session, subjects were asked to return on 4 separate days to perform handgrip exercise with and without GTN administration (i.e. two control (CON) trials and two GTN trials). For the determination of resting heart rate (HR) and blood pressure (BP), subjects were asked to lie down in the supine position for 20 min with their dominant arm outstretched to approximately 90º. All testing was performed in a quiet, temperature‐controlled room (22–24ºC) with the lighting reduced for the optimal visualization of the vessel image and blood velocity profile.

Each dynamic exercise trial consisted of an initial baseline image, including the longitudinal view of the vessel and the blood velocity profile, that was recorded for 30 s followed by 6 min of forearm contractions at 5% of the subjects’ MVC. Data from the two trials in each condition were then averaged and used for statistical analysis. The load was placed at the end of a custom‐built pulley system attached to a handgrip dynamometer and designed to move the load 75 mm. Subjects were instructed to contract and relax the forearm on each beat of the metronome, resulting in a contraction frequency of 30 contractions/min. Each dynamic exercise trial was performed on separate days for the CON and GTN conditions. For the GTN condition, subjects were administered 0.4 mg of GTN sublingually following the 30 s baseline. Brachial artery diameter and blood velocity were measured continuously until the brachial artery diameter approximated the new steady‐state value, which was recorded during the familiarization session. Once the increase in arterial diameter steady‐state was achieved, subjects were instructed to begin the handgrip exercise protocol and both the brachial artery diameter and blood velocity were measured for the next 6 min.

### 2.5 Experimental measurements

HR and BP were measured continuously throughout baseline and exercise using an automated finger plethysmography system (Finometer Model 1, Finapres Medical Systems). Total peripheral resistance (TPR) was calculated according to the equation;

TPR=MAP/Q

where MAP is the mean arterial pressure and Q is the cardiac output (Tschakovsky et al., 1996). Forearm vascular conductance (FVC) was calculated using the following equation;

FVC=FBF/MAP

where FBF is the mean forearm blood flow.

Cardiac output was calculated from the HR and SV data collected from the automated finger plethysmography system (Finometer Model 1, Finapres Medical Systems).

Longitudinal images of the brachial artery were obtained using B‐mode two‐dimensional ultrasonography using a multiple‐frequency linear array probe (Model L8‐2; center frequency of 7.5 MHz) attached to an ultrasound system operating in Duplex mode (z.one ultra, Zonare Medical Systems Inc.). The ultrasound probe was placed approximately 3–5 cm proximal to the antecubital fossa on the medial aspect of the upper arm and was secured in place using a custom‐built articulating arm and clamp once the optimal image was found. Recording of the images for measuring vessel diameter and the Doppler ultrasound measurements of blood velocity were achieved using the same transducer operating simultaneously in 2D echo and pulse wave Doppler modes. Blood velocity measurements were obtained with the transducer positioned to maintain an angle of insonation of ≤60° relative to the position of the artery. The gate was adjusted to the width of the artery to ensure that the sample volume included the near and far wall borders and remained centered in the lumen of the vessel.

Frequency‐domain multi‐distance near‐infrared spectroscopy was used to continuously monitor the local muscle oxygenation of the forearm muscles (NIRS; Oxiplex TS, ISS). The theory and application of the use of NIRS have been previously described (Ellsworth et al., 1995). Briefly, a lightweight probe, which is connected to laser diodes and photomultiplier by optical fibers, utilizes two rows of light‐emitting fibers and one detector fiber bundle was placed on the surface of the skin. The eight laser diodes, four of which operate at a wavelength of 690 nm and the other four at 828 nm, are separated by 2.0, 2.5, 3.0, and 3.5 cm for each of the wavelengths. This arrangement of emitting diodes and detector bundle allows for the continuous measurement of the absolute concentrations (μM) of oxygenated heme (oxy‐[heme]) and deoxygenated heme (deoxy‐[heme]). The concentration of total heme (total [heme]) can be calculated as the sum of oxy‐[heme] and deoxy‐[heme]. Since the absorption spectra of myoglobin are similar to that of hemoglobin, the NIRS signals represent both vascular hemoglobin and muscle myoglobin (Ferreira et al., 2005). The NIRS probe was placed on the surface of the skin over the flexor carpi radialis, palmaris longus, and flexor carpi ulnaris muscles and secured to the forearm using a Velcro strap. The probe was covered with an optically dense fabric and secured in position using self‐adhering tape (MedCo). The position of the NIRS probe was marked after each trial using a permanent pen in an effort to reposition the NIRS probe over the same sampling site for each trial. The NIRS system was calibrated prior to each data collection session according to the instructions and specifications provided by the manufacturer after allowing the instrument to warm‐up for no <20 min. NIRS data were collected at a sampling frequency of 2 Hz and stored on a computer for off‐line analysis at a later time.

### 2.6 Data processing

The image of the brachial artery and the corresponding blood velocity profiles were recorded at a sampling frequency of 30 Hz using a digital imaging frame grabber (DVI2USB 3.0, Epiphan Systems Inc.) and acquisition software system (Cardiovascular Suite 2.1, Quipu srl). The video files (vessel wall and blood velocity recordings) were stored on a computer for offline analysis at a later time. Forearm blood flow (FBF) was calculated as:

$$\text{FBF(mL/min)}\;=\;\text{MBV}\;\times \;\pi r^{2}\;\times \;60\;s.$$

where FBF is the mean forearm blood flow and MBV is the mean blood velocity.

The arterial waveform from finger plethysmography was filtered using an analog‐to‐digital converter (PowerLab 8/35, ADInstruments) and analyzed for systolic, diastolic, and mean arterial pressures using LabChart (ADInstruments) The analog signal was collected at 1000 Hz and down‐sampled by a factor of 1000 in order to generate 1‐s bins.

The frame‐by‐frame data from the vessel diameter and blood velocity analysis were collected at 30 Hz and were reduced into 1‐s averages using an Excel Macro (Microsoft). Antegrade and retrograde blood velocities were recorded into two columns by the acquisition software (Cardiovascular Suite 2.1, Quipu srl). Mean blood velocity was calculated as the difference between the antegrade and retrograde velocities at each time point. Data from the Doppler video analysis were time‐aligned with the central hemodynamic variables. For CON and GTN conditions, the NIRS data were ensemble‐averaged for the two trials resulting in a single response for each subject and subsequently averaged into 10‐s bins for statistical analysis. NIRS data were not first reduced into 1‐s averages due to the lower sampling frequency (2 Hz). The difference (Δ) from rest (prior to GTN administration) was calculated for total [heme], oxy‐[heme], and deoxy‐[heme] for comparison between CON and GTN conditions.

For each subject, responses from the CON and GTN trials for central hemodynamics and blood flow were ensemble‐averaged using the 1‐s bins. Subsequently, a 10 s average was calculated each for time point and responses were compared every 10 s for the first 2 min of exercise and every 60 s after until the end of the exercise.

### 2.7 Statistical analysis

A two‐way analysis of variance (ANOVA) with repeated measures (condition, time) was conducted to determine differences in muscle blood flow (brachial artery diameter, antegrade velocity, and retrograde velocity) between CON and GTN groups, as well as differences in NIRS (total [heme], oxy‐[heme], deoxy‐[heme]) data. A Tukey's post hoc test was used, if a significant F‐ratio was reported as a result of the ANOVA analysis. All central hemodynamic variables (HR, SV, systolic pressure, diastolic pressure, and TPR) were analyzed using a two‐way ANOVA with repeated measures (condition, time) and a Tukey's post hoc test was used if a significant *F*‐ratio was reported as a result of the ANOVA analysis. Statistical significance was set a priori at *p* ≤ 0.05. Data were analyzed with the use of Sigma Plot software (SigmaPlot 13.0, Systat Software, Inc.). All data are presented as the mean ± standard deviations (SD).

## 3 RESULTS

Nine subjects were recruited to participate in this study. One subject was excluded prior to data collection, as clearly defined artery wall borders were unattainable after multiple attempts. Thus, the data from eight healthy subjects (29 ± 9 years) are reported. All subjects were normotensive (SBP: 118 ± 15 mmHg; DBP: 52 ± 9 mmHg). The group mean handgrip MVC for the eight subjects was 53.9 ± 9.6 kg. Subjects performed dynamic contractions at 5% of MVC; thus, the mean load for the handgrip exercise was 2.7 ± 0.4 kg.

### 3.1 Forearm blood flow responses

**FIGURE 1:**
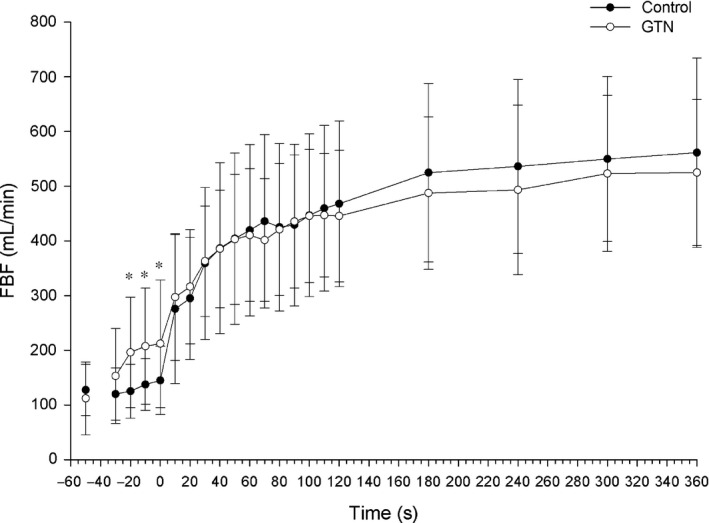
Forearm blood flow. Forearm blood flow averaged at 10‐sec intervals for the first 2 min of exercise and every minute after until the end of the exercise protocol. Time = 0 seconds represents the start of contractions. Time point (−50) was assessed as the 30 s average of resting data for Control and prior to GTN administration. *indicates significance at *p* < 0.05. Two‐way ANOVA with repeated measures (time, condition) followed by Tukey's posttest

Forearm blood flow, brachial artery diameter, and mean blood velocity were not significantly different between conditions, at rest, prior to exercise and GTN administration. This investigation was designed to induce a significant increase in brachial artery diameter by GTN administration. As intended by experimental design, there was a significant main effect for time and condition (*F*_(1,7)_ = 5.44, *p* < 0.001; *F*_(1,7)_ = 161.23, *p* < 0.001, respectively), as well as a significant interaction for time × condition (*F*_(1,7)_ = 5.03, *p* < 0.001). Brachial artery diameter was significantly greater at baseline following GTN administration compared to CON (CON, 0.49 ± 0.07 cm; GTN, 0.56 ± 0.07 cm, *p* < 0.05; Figure 1).

**FIGURE 2:**
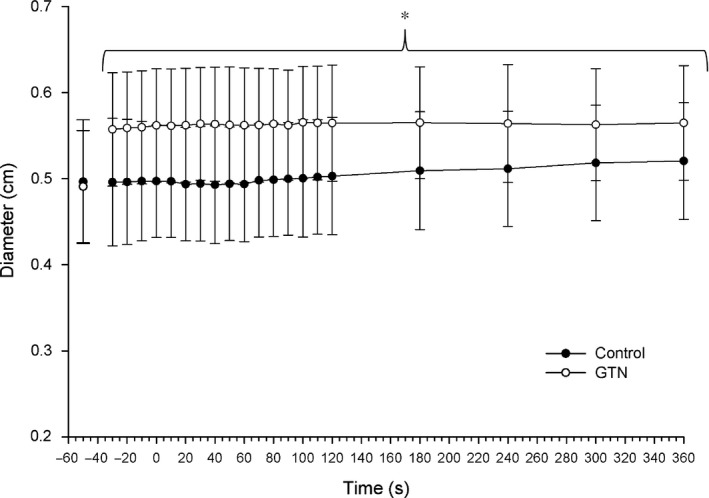
Changes in brachial artery diameter. Brachial artery diameter assessed at multiple time points throughout exercise. Time point (−50) was assessed as the 30 s average of resting data for Control and prior to GTN administration. *indicates significance at *p* < 0.05. Two‐way ANOVA with repeated measure (time, condition) followed by Tukey's posttest

For FBF, the results of the ANOVA revealed that there was a significant interaction effect for time × condition (*F*_(1,7)_ = 2.94, *p* < 0.001). Further post hoc analysis revealed that for the 30 s prior to the onset of exercise, FBF was significantly higher following GTN administration compared to CON (*p* < 0.05). However, there was no significant difference in FBF after the onset of muscle contractions between CON and GTN conditions (Figure 2). There was no main effect for condition; however, there was a significant main effect for time (*F*_(1,7)_ = 42.21, *p* < 0.001), indicating that there was a significant increase in FBF throughout the exercise.

**FIGURE 3:**
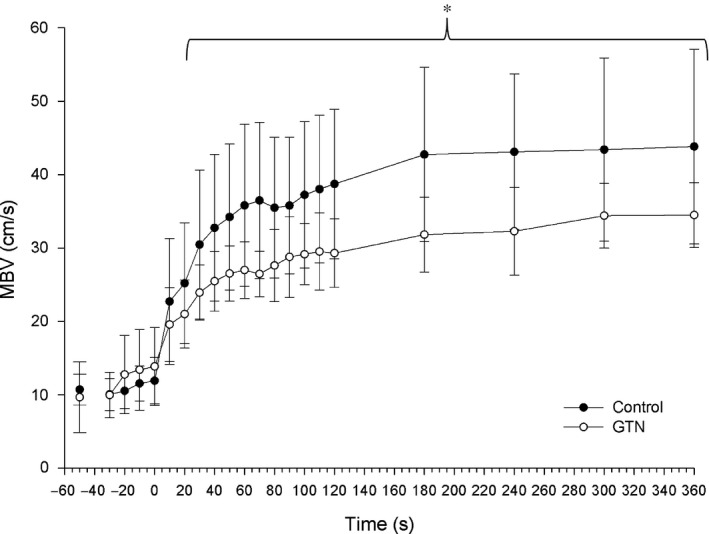
Mean blood velocity. Mean blood velocity averaged at 10‐sec intervals for the first 2 min of exercise and every minute after until the end of the exercise protocol. Time point (−50) was assessed as the 30 s average of resting data for Control and prior to GTN administration. *indicates significance at *p* < 0.05. Two‐way ANOVA with repeated measures (time, condition) followed by Tukey's posttest

The results of the ANOVA of mean blood velocity (MBV) indicated that there was a main effect for time (*F*_(1,7)_ = 42.49, *p* < 0.001) and condition (*F*_(1,7)_ = 5.71, *p* = 0.048), as well as a significant time × condition interaction (*F*_(1,7)_ = 7.82, *p* < 0.001). There was no significant difference between CON and GTN conditions at baseline prior to the onset of exercise (CON, 11.92 ± 3.17 cm/s; GTN, 13.85 ± 5.30 cm/s, *p* > 0.05); however, MBV was higher in CON compared to GTN (*p* < 0.05) following the first 40 s of handgrip exercise (Figure 3).

**FIGURE 4:**
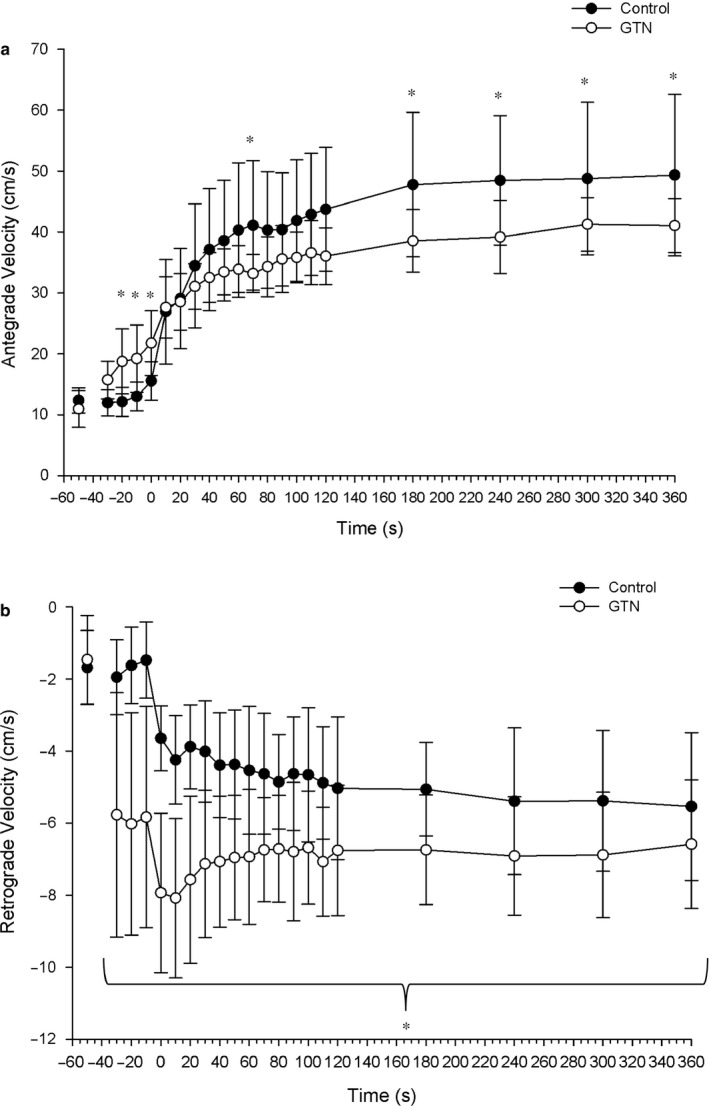
Antegrade and retrograde blood velocities. Antegrade velocity (a) was significantly higher prior to exercise in the GTN condition, but lower after 180 s of exercise. Retrograde blood velocity was significantly greater at all time points in the GTN condition. *indicates significance at *p* < 0.05. Two‐way ANOVA with repeated measures (time, condition) followed by Tukey’s posttest.

Since MBV was significantly higher in CON than GTN for most of the exercise, MBV was further analyzed by comparing antegrade and retrograde velocities in order to identify any differences in directional flow. The ANOVA revealed a significant main effect for time (*F*_(1,7)_ = 41.14, *p* < 0.001), as well as a time × condition interaction effect (*F*_(1,7)_ = 10.45, *p* < 0.001) for antegrade blood velocity. Antegrade blood velocities were significantly greater during exercise than resting values in both GTN and CON. Antegrade blood velocity was significantly higher in GTN for the 20 s prior to exercise; however, after 2 min of dynamic muscle contractions, antegrade blood velocity was significantly lower in GTN compared to CON. Retrograde blood velocity showed a significant main effect for time (*F*_(1_,_7)_ = 31.21, *p* < 0.001) and condition (*F*_(1,7)_ = 38.54, *p* < 0.001), as well as an interaction effect for time × condition (*F*_(1,7)_ = 4.46, *p* < 0.001). Retrograde blood velocity was significantly greater following GTN administration at all time points compared to CON (*p* < 0.05; Figure 4).

**FIGURE 5:**
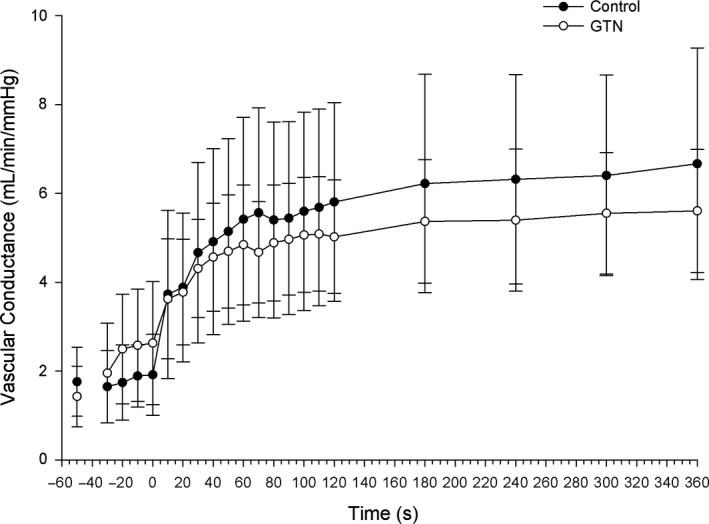
Vascular conductance. There were no differences between conditions in vascular conductance throughout the duration of exercise

Forearm vascular conductance (FVC) showed a main effect for time (*F*_(1,7)_ = 37.58, *p* < 0.001), but there was no main effect for condition or time x condition interaction, indicating that there was an increase in FVC with handgrip exercise but there was no difference between CON and GTN conditions (Figure 5).

### 3.2 Central cardiovascular responses

For mean arterial pressure (MAP), there was a significant main effect for time (*F*_(1,7)_ = 23.20, *p* < 0.001). MAP increased from 77 ± 9 mmHg at rest in CON to 87 ± 11 mmHg at end‐exercise. MAP increased from 80 ± 8 mmHg at rest in GTN to 94 ± 6 mmHg at end‐exercise. However, there was no difference in MAP between CON and GTN conditions. Similarly, a significant main effect for time (*F*_(1,7)_ = 19.18, *p* < 0.001; *F*_(1,7)_ = 24.64, *p* < 0.001, respectively) was observed for systolic and diastolic blood pressure (SBP and DBP) such that SBP and DBP increased at the onset of exercise and continued to increase throughout the duration of the exercise. However, there was no difference between CON and GTN conditions for either SBP or DBP.

The results of the ANOVA for cardiac output (Q) indicated that there was a main effect for time (*F*_(1,7)_ = 5.86, *p* < 0.001) but there was no main effect for condition or time × condition interaction, indicating that there was a slight increase in Q with handgrip exercise but there was no difference between CON and GTN conditions. For both heart rate (HR) and stroke volume (SV), the results of the ANOVA indicated that there was a main effect for time (HR: *F*_(1,7)_ = 7.25, *p* < 0.001; SV: *F*_(1,7)_ = 3.11, *p* < 0.001), a main effect for condition (HR: *F*_(1,7)_ = 7.13, *p* = 0.032; SV: *F*_(1,7)_ = 12.04, *p* = 0.01) and interaction effects for time × condition (HR: *F*_(1,7)_ = 2.29, *p* = 0.02; SV: *F*_(1,7)_ = 1.74, *p* = 0.03). The results of the post hoc multiple comparison test indicated that HR increased over time in both conditions, but HR was greater in GTN (73 ± 8 bpm) at the onset of muscle contractions compared to CON (61 ± 8 bpm). SV decreased significantly following GTN administration (CON, 123.0 ± 20.7 ml; GTN, 102.8 ± 10.9 ml; *p* < 0.05) and remained lower throughout exercise (CON, 130.5 ± 22.9 ml; GTN, 100.6 ± 20.5 ml; *p* < 0.05).

Total peripheral resistance (TPR) showed a main effect for time (*F*_(1,7)_ = 3.75, *p* < 0.001) and a significant interaction for time x condition (*F*_(1,7)_ = 3.15, *p* < 0.001). TPR was significantly greater following GTN administration compared to CON, but only at 90, 240, 300, and 360 s of exercise (*p* < 0.05).

### 3.3 Near‐infrared spectroscopy

**FIGURE 6:**
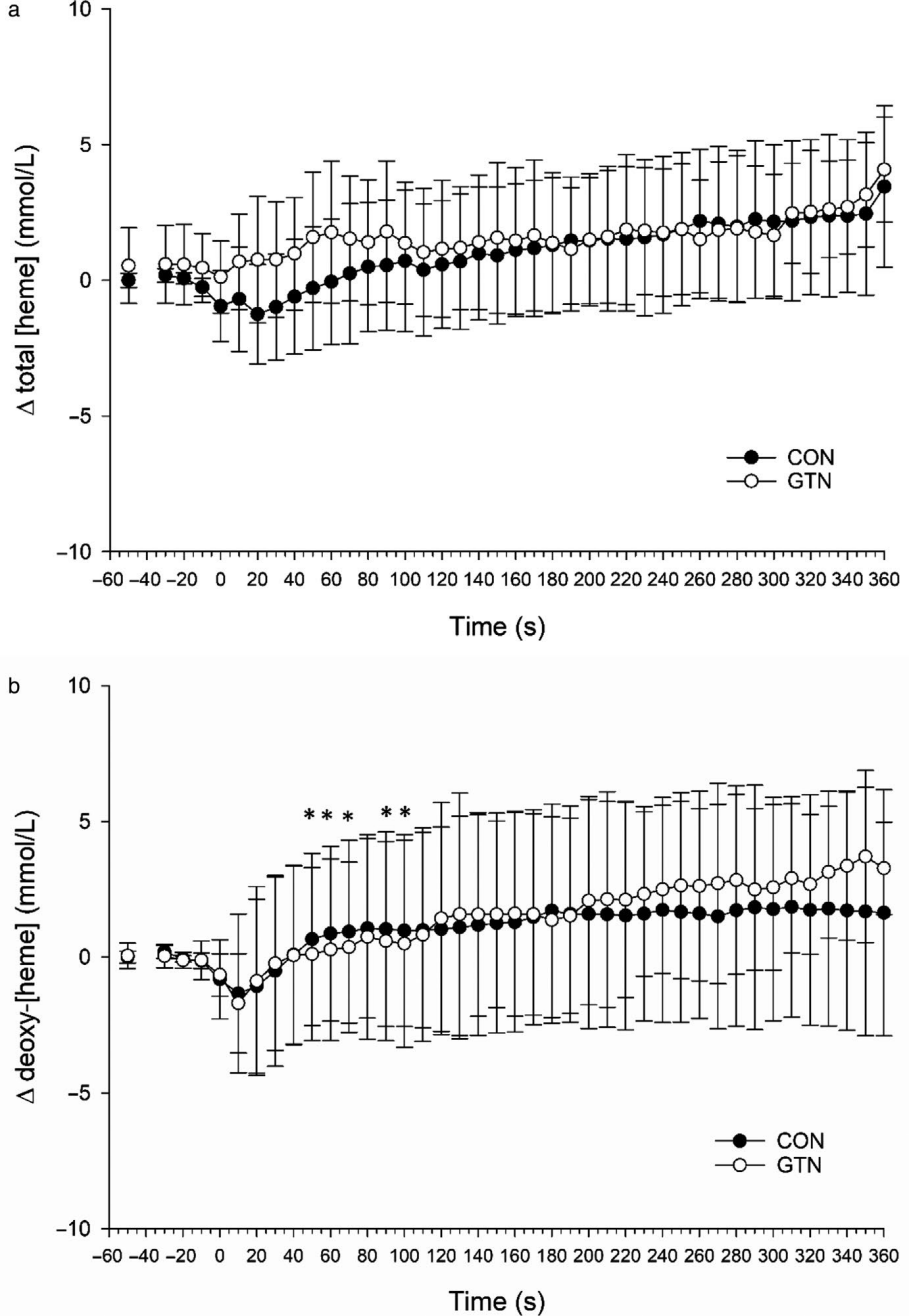
Change in total hemoglobin concentration and de‐oxygenated hemoglobin as assessed by nearinfrared spectroscopy (NIRS). There were no differences in [THC] (a) between conditions. *represents difference between conditions for [HHb], *p* < 0.05 (b). Two‐way ANOVA with repeated measures (condition, time) followed by Tukey's posttest

Deoxy‐[heme] showed a significant increase from rest to the end of exercise (CON = 3.37 ± 3.31 µM ΔRest; GTN = 3.54 ± 4.48 µM ΔRest). [HHb] was significantly lower in GTN compared to CON at 50, 60, 70, 90, and 100 s of exercise. There was no significant difference in either total [heme] or oxy‐[heme] between CON and GTN conditions. Changes in total [heme] and deoxy‐[heme] throughout exercise are presented in Figure 6.

## 4 DISCUSSION

The purpose of this study was to examine the blood flow response following vasodilation prior to the onset of handgrip exercise using GTN administration. It was hypothesized that FBF would be elevated at rest due to GTN administration and that FBF would further increase above control values with the addition of handgrip exercise. The primary finding of this study was that in spite of an increase of 12% in brachial artery diameter and a significant increase in FBF following GTN administration at rest, there was no significant difference in FBF between GTN and control conditions. These findings indicate that FBF was well matched to the demands of exercise and that the initial over‐perfusion seen in the GTN condition at rest did not persist throughout the exercise.

### 4.1 Forearm blood flow

There was no significant difference in muscle blood flow during dynamic handgrip exercise following GTN administration, in spite of a significant increase in brachial artery diameter and FBF prior to the onset of exercise. These results are in agreement with Terwood et al. (2018), who reported similar changes in MBF following sodium nitroprusside (SNP) infusion during low‐intensity, rhythmic handgrip exercise. However, these authors did not determine artery diameter and blood velocities; whereas the present investigation showed significant changes to both antegrade and retrograde blood velocities following GTN administration. Additionally, brachial artery diameter was significantly greater at every time point following GTN administration; however, MBV was significantly lower after 40 s of exercise which could explain the lack of differences seen in forearm muscle blood flow. Although arterial diameter was greater with GTN administration at all time points, there were no further changes in brachial arterial diameter before or during dynamic, rhythmic handgrip exercise. In contrast to our original hypothesis, brachial artery diameter and FBF did not increase any further with GTN, indicating that the blood flow response in the conduit artery may not be an accurate reflection of the blood flow response in the microvasculature. This finding emphasizes the importance of measuring changes in conduit artery diameters and blood velocities under conditions where changes in arterial diameter may provide for alternative interpretations of the overall blood flow response as a result of the experimental conditions.

It is also likely that a hierarchy of vascular control exists within the vasculature that differs considerably between the conduit artery and the microvasculature. The results of this study indicated that both the conduit artery diameter and MBV were significantly increased in a NO‐dependent manner prior to the onset of exercise. The findings of previous research have demonstrated that endothelial shear stress and NO are primary control mechanisms for vascular tone within the conduit artery (see review Mortensen & Saltin, 2014); whereas, some evidence suggests that oxygen tension may play a role in microvascular blood flow regulation (Ferreira et al., 2005). However, given the similar changes in total [heme] and deoxy‐[heme] observed in the present study, it appears that within the microvasculature, NO plays a minimal role in matching oxygen delivery to oxygen demand. Had NO played a significant role in microvascular blood flow, total [heme], which reflects the amount of hemoglobin under the region of interrogation by the probe, would have been greater following GTN administration, owing to greater vasodilation of the microvasculature, compared to CON. Since the intensity of contractions was similar between conditions, it is possible that the oxygen tension between conditions was similar thereby providing evidence for the potential for different regulatory factors of vascular tone between the macro‐ and micro‐vasculature.

Moreover, when MBV was examined in terms of antegrade and retrograde blood velocities, a significant increase in retrograde velocity was observed following GTN administration compared to CON that persisted throughout the dynamic exercise. The observed retrograde velocities in CON were due to the contraction of skeletal muscle (Walløe & Wesche, 1988); however, the magnitude of retrograde blood velocity was much greater in the GTN condition during exercise. Additionally, antegrade velocity was increased at the onset of dynamic contractions; however, antegrade velocity was lower following GTN administration compared to CON. Thus, it appears GTN administration led to a reduction in MBV as a result of a significant increase in retrograde blood velocity at rest and during dynamic forearm exercise. These findings are in contrast to Shepherd et al. (Shepherd et al., 2016) which found an additive effect of prior vasodilation to the muscle blood flow response to forearm contractions. These authors continuously infused ATP, a known vasodilator, prior to and throughout the low‐intensity exercise, with the intent of dissociating oxygen delivery from oxygen demand. However, they concluded that some unexplained components of the exercise hyperemic response were independent of oxygen demand (Shepherd et al., 2016). In contrast, the results of the present investigation would argue that regardless of the initial over‐perfusion that was seen prior to exercise, FBF appears to be matched to the oxygen demand of the exercising muscle within the first 10 s of the onset of exercise. Additionally, (Shepherd et al., 2016) did not report brachial artery diameter and blood velocity; therefore, it is difficult to identify the mechanism that resulted in a significant increase in FBF and vascular conductance. In the present investigation, it is evident that changes in blood velocity with GTN administration played a significant role in the overall MBF response to exercise. The results of the present investigation demonstrated a significant increase in retrograde blood velocity following the administration of GTN that persisted throughout the forearm exercise. The appearance of retrograde blood velocity represents a brief, transient change in the direction of blood flow toward the heart that has been associated with an increase in downstream vascular resistance (Walløe & Wesche, 1988). Unlike the present investigation, (Shepherd et al., 2016) selected ATP infusion to provide robust vasodilation without affecting systemic hemodynamics. However, ATP has been shown to have not only vasodilatory properties, but has also been shown to override sympathetic vasoconstrictor activity (Radegran & Saltin, 1998; Ranadive et al., 2019). GTN has not been shown to have sympatholytic properties. In fact, 0.4 mg dosage of GTN has been shown to increase muscle sympathetic nerve activity, albeit in a subgroup of hypertensive patients (Ghiadoni et al., 2019) and therefore it is possible that the sympathetic vasoconstrictor response in the present investigation remained intact. This is evidenced by the greater retrograde velocity following GTN administration compared to the CON condition. Therefore, it is possible that although GTN provided robust vasodilation within the conduit artery, there was a greater sympathetic outflow to the exercising muscle in order to correct the over‐perfusion seen prior to exercise.

Our results are in agreement, at least in part, with Hamann et al. (2003) which showed a decrease in hindlimb blood flow at the onset of exercise under prior vasodilation, albeit this was shown in the dog hindlimb model. Additionally, (MacDonald et al., 1998) have demonstrated that leg blood flow was not different between upright and supine exercise despite significantly different femoral artery diameters. Additionally, MAP was lower in the supine group, as expected and therefore perfusion pressure was lower. These authors concluded that the similar leg blood flow response between conditions, in spite of a significant decrease in MAP, was due in large part to local vasodilation that occurred within the active skeletal muscle in the supine position. Similar to their results, FBF was not different between CON and GTN conditions in the current investigation despite a significantly larger arterial diameter following GTN administration. However, both the CON and GTN conditions were completed in the supine position and therefore, there was no difference in MAP or perfusion pressure between the CON and GTN conditions. This can be evidenced, at least in part, by FVC measurements. There were no significant differences in FVC between CON and GTN conditions. This leads these authors to believe that perfusion pressure did not have a significant impact on MBF or blood velocity measurements. Combining the results of this study with the results of MacDonald et al. (1998), it is possible that local vasodilation occurred to the same extent in the CON and GTN conditions, despite differences in conduit artery diameter, further strengthening the notion that a control hierarchy exists between the macro‐ and micro‐vasculature.

### 4.2 Central hemodynamics/mean arterial pressure

The perfusion pressures between conditions in the current investigation appear to be the same, as MAP and cardiac output were not different between conditions. Perfusion pressure has been shown to regulate muscle blood flow during exercise as (Tschakovsky et al., 1996) demonstrated that increasing perfusion pressure prior to muscle contraction increases muscle blood flow following that contraction. Relevant to the present investigation, it is possible that the similar perfusion pressures between GTN and CON can explain the similar MBF between conditions as well. Interestingly, SV was significantly lower following GTN administration and was matched by an elevation in HR to maintain cardiac output. Additionally, the decrease in SV was maintained as the exercise progressed. It is possible that the initial decrease in SV seen with GTN administration provoked an increase in sympathetic nerve activity leading to an increase in HR and sympathetic vasoconstriction in the periphery. Hughes et al. (2017) have recently shown that SNS activation, via the cold pressor test, can attenuate contraction‐induced rapid vasodilation, and therefore blood flow in the human leg. If an increase in HR is indicative of an increase in SNS activity, then it is possible that any increase in FBF that would have been seen in the GTN condition due to the large increase in arterial diameter was offset by an increase in muscle sympathetic nerve activity (MSNA) downstream of the feed artery. An increase in MSNA downstream of the feed artery may explain, at least in part, the greater retrograde blood velocities observed following GTN administration (Valic et al., 2002). The extent that GTN administration altered vascular tone within the microvasculature relative to the conduit artery is not known but may prove important in understanding the link between local vasodilation and sympathetic vasoconstriction.

It should be noted that GTN has been shown to be a selective dilator of both the venous system and muscular arteries (Omar et al., 2015). Thus, the effect of GTN should not be limited to only the arterial system. It is possible that venous pooling occurred; therefore, the increased retrograde and lower stroke volume observed in the present investigation may be explained by venous dilation and pooling in the venous system (Kelly et al., 1990). Kelly and associates also found a decrease in MAP following GTN administration, which they have associated with the decrease in SV (Kelly et al., 1990). However, the present investigation found no difference in MAP between GTN and CON conditions. Therefore, these authors believe that a GTN‐induced dilation of venous circulation did not have a significant impact on retrograde blood velocity.

### 4.3 Near‐infrared spectroscopy

We originally hypothesized that GTN administration would systemically vasodilate both the conduit artery and the microvasculature, thereby increasing forearm blood volume; therefore, we would have expected to see a higher total [heme] content, which can be interpreted as changes in blood volume within the tissue (Van Beekvelt et al., 2001). As shown in Figure 6a and 6b, there was no difference in either total [heme] or deoxy‐[heme] between GTN and CON conditions. The more interesting point is that total [heme] was not different between the two conditions. Because there was no difference between CON and GTN in the present investigation, it can be inferred that the blood volume within the interrogation volume was similar between the conditions. Again, this could mean that within the microvasculature, vascular tone had recovered in an attempt to correct the overperfusion seen within the feed artery. This is evidenced once again by the significantly greater retrograde blood velocity seen following GTN administration before and during exercise. Retrograde blood velocity represents blood flow toward the heart and can be used as a representation of downstream vascular resistance (Walløe & Wesche, 1988). Therefore, one might expect to see differences in total [heme] to represent an increased microvascular blood flow. The greater retrograde blood velocity reported in the current investigation supports the idea that vasoconstriction occurred within the microvasculature in order to correct the overperfusion caused by GTN administration resulting in significant conduit artery vasodilation.

### 4.4 Limitations

The present study used a contraction intensity of 5% MVC, which could possibly explain the lack of differences in FBF in between groups. In the human model, evidence has shown that NO plays a contraction intensity‐dependent role in the muscle blood flow response to exercise (Casey et al., 2015; Kelly et al., 1990; Omar et al., 2015). Richards et al. (Richards et al., 2018) found that FBF did not differ significantly between ${\text{NO}}_{3}^{‐}$ supplementation and control until contractions occurred at intensities greater than 5% MVC. Unlike Shepherd et al. (2016), which utilized a contraction intensity of 20% MVC, participants in the present study investigation, were unable to adhere to the exercise protocol for the entire duration (6 min). Perhaps changing the duty cycle (30 contractions/min) or the length of the exercise protocol would allow for higher contraction intensity in future research. Thus, if muscle contractions had been performed at a higher intensity, it is possible that we could have seen a difference in the blood flow responses to GTN and control conditions. However, the authors of the present investigation wanted the exercise intensity to play a very limited role in brachial artery diameter, and so a very low contraction intensity was utilized. Additionally, (Radegran & Saltin, 1998) found that NO was responsible for increasing FBF at rest and during post‐exercise recovery, but had no effect on the exercise hyperemia regardless of exercise intensity. The findings of the present investigation would seem to support this idea.

Last, oral administration of GTN will result in a systemic hemodynamic effect, as opposed to local vasodilator infusions, as reported in Shepherd et al. (2016). This would result in a greater bioavailability of NO in local vasodilator infusion compared to orally administered GTN (Limberg et al., 2020). This may help to explain the differences in MBF with the superimposition of exercise in the present investigation compared to the results of Shepherd and associates (Shepherd et al., 2016). It is possible that a greater bioavailability of NO resulted in significant increases in FVC, as shown by Shepherd et al. (Shepherd et al., 2016), but not by the present investigation. Therefore, it is possible that the lack of differences in MBF throughout exercise in the present investigation could be the result of a decreased bioavailability of NO, particularly at the level of the microvasculature.

### 4.5 Conclusion

This study attempted to dissociate oxygen delivery (i.e. MBF) from oxygen demand using prior administration of GTN to induce significant vasodilation of the brachial artery. We initially hypothesized that MBF would be higher at rest following the administration of GTN due to an increase in brachial arterial diameter and that the addition of handgrip exercise would lead to further vasodilation and a further increase in FBF. In spite of a 12% increase in brachial artery diameter and a concomitant increase in resting FBF following GTN administration, FBF was not different with the addition of dynamic exercise from the control condition, suggesting that prior vasodilation of the conduit artery does not play a significant role in the matching of oxygen delivery to the metabolic demand of the contracting muscle during low‐intensity exercise.

## CONFLICT OF INTEREST

None declared.

## AUTHOR CONTRIBUTIONS

T.R. and B.S. conceived and designed the study. T.R. performed data acquisition. T.R., J.L., J.T., and B.S. contributed to the analysis and interpretation of the data. T.R. and B.S. drafted the paper. J.L., J.T., and B.S. provided feedback on the paper. All authors approved the final version of the manuscript and agree to be accountable for all aspects of the work in ensuring that questions related to the accuracy or integrity of any part of the work are appropriately investigated and resolved. All persons designated as authors qualify for authorship, and all those who qualify for authorship are listed.
